# Choline Kinase Alpha as an Androgen Receptor Chaperone and Prostate Cancer Therapeutic Target

**DOI:** 10.1093/jnci/djv371

**Published:** 2015-12-11

**Authors:** Mohammad Asim, Charles E. Massie, Folake Orafidiya, Nelma Pértega-Gomes, Anne Y. Warren, Mohsen Esmaeili, Luke A. Selth, Heather I. Zecchini, Katarina Luko, Arham Qureshi, Ajoeb Baridi, Suraj Menon, Basetti Madhu, Carlos Escriu, Scott Lyons, Sarah L. Vowler, Vincent R. Zecchini, Greg Shaw, Wiebke Hessenkemper, Roslin Russell, Hisham Mohammed, Niki Stefanos, Andy G. Lynch, Elena Grigorenko, Clive D’Santos, Chris Taylor, Alastair Lamb, Rouchelle Sriranjan, Jiali Yang, Rory Stark, Scott M. Dehm, Paul S. Rennie, Jason S. Carroll, John R. Griffiths, Simon Tavaré, Ian G. Mills, Iain J. McEwan, Aria Baniahmad, Wayne D. Tilley, David E. Neal

**Affiliations:** **Affiliations of authors:**Cancer Research UK Cambridge Institute, University of Cambridge, Cambridge, UK (MA, CEM, NP, HIZ, AQ, AB, SM, BM, CE, SL, SW, VRZ, GS, RR, HM, AGL, CD, CT, AL, RS, JY, RS, JSC, JRG, ST, DEN); School of Medical Sciences, University of Aberdeen, Aberdeen, UK (FO, IJM); Department of Pathology, Addenbrooke’s Hospital, Cambridge, UK (AYW, NS); Institute of Human Genetics, Jena University Hospital, Jena, Germany (ME, KL, WH, AB); Dame Roma Mitchell Cancer Research Laboratories, School of Medicine, Faculty of Health Sciences, University of Adelaide, Australia (LAS, WDT); Freemasons Foundation Centre for Men’s Health, School of Medicine, Faculty of Health Sciences, University of Adelaide, Australia (LAS, WDT); Diatherix, Huntsville, AL (EG); Masonic Cancer Center, University of Minnesota, Minneapolis, MN (SMD); The Vancouver Prostate Centre, Department of Urologic Sciences, University of British Columbia, Vancouver BC, Canada (PSR); Centre for Molecular Medicine Norway, Nordic EMBL Partnership, University of Oslo and Oslo University Hospital, Oslo, Norway (IGM); Department of Cancer Prevention, Institute of Cancer Research and Department of Urology, Oslo University Hospital, Oslo, Norway (IGM); Prostate Cancer UK/Movember Centre of Excellence, Queens University, Belfast, UK (IGM); Department of Oncology, Addenbrooke’s Hospital, Cambridge, UK (MA, DEN).

## Abstract

**Background:**

The androgen receptor (AR) is a major drug target in prostate cancer (PCa). We profiled the AR-regulated kinome to identify clinically relevant and druggable effectors of AR signaling.

**Methods:**

Using genome-wide approaches, we interrogated all AR regulated kinases. Among these, choline kinase alpha (CHKA) expression was evaluated in benign (n = 195), prostatic intraepithelial neoplasia (PIN) (n = 153) and prostate cancer (PCa) lesions (n = 359). We interrogated how CHKA regulates AR signaling using biochemical assays and investigated androgen regulation of CHKA expression in men with PCa, both untreated (n = 20) and treated with an androgen biosynthesis inhibitor degarelix (n = 27). We studied the effect of CHKA inhibition on the PCa transcriptome using RNA sequencing and tested the effect of CHKA inhibition on cell growth, clonogenic survival and invasion. Tumor xenografts (n = 6 per group) were generated in mice using genetically engineered prostate cancer cells with inducible CHKA knockdown. Data were analyzed with χ^2^ tests, Cox regression analysis, and Kaplan-Meier methods. All statistical tests were two-sided.

**Results:**

CHKA expression was shown to be androgen regulated in cell lines, xenografts, and human tissue (log fold change from 6.75 to 6.59, *P* = .002) and was positively associated with tumor stage. CHKA binds directly to the ligand-binding domain (LBD) of AR, enhancing its stability. As such, CHKA is the first kinase identified as an AR chaperone. Inhibition of CHKA repressed the AR transcriptional program including pathways enriched for regulation of protein folding, decreased AR protein levels, and inhibited the growth of PCa cell lines, human PCa explants, and tumor xenografts.

**Conclusions:**

CHKA can act as an AR chaperone, providing, to our knowledge, the first evidence for kinases as molecular chaperones, making CHKA both a marker of tumor progression and a potential therapeutic target for PCa.

Prostate cancer (PCa) is a major cause of cancer-related deaths worldwide (1). The androgen receptor (AR) is a ligand-inducible transcription factor of the nuclear hormone receptor superfamily that plays a critical role in tumor initiation, growth, and progression of PCa (2,3). Hence, therapies targeting the AR signaling axis provide an effective first-line treatment for advanced PCa (4,5). As with many other cancer types, resistance to therapy occurs in PCa in the form of progression to advanced castration-resistant prostate cancer (CRPC) (6,7) and is accompanied by reactivation or maintenance of AR signaling, which triggers a unique AR transcriptome (8). Multiple direct mechanisms can stimulate AR signaling in advanced PCa, including amplification, gain-of-function mutations in the AR gene/androgen signaling pathway (9), and constitutively active AR splice variants such as AR-V7 (10,11). Indirect mechanisms driving elevation of AR protein expression in PCa include the upregulation of heat shock proteins (HSPs) that act as chaperones for AR. HSPs interact with the LBD of AR and promote its stability, folding, and activation. Consistent with this, targeting of HSPs in preclinical models inhibits AR function and tumor growth (12,13). In addition, we and others have shown the importance of kinases in regulating AR function and PCa progression (14–16).

These diverse resistance mechanisms highlight the reliance of PCa on the maintenance of AR signaling, which controls a number of cellular pathways including metabolic fuelling of tumor growth (17), progression through cell cycle checkpoints (18), promotion of metastatic phenotypes (19), and DNA damage repair (20,21). Moreover, a well-established feature of AR signaling in PCa is the existence of multiple feedback and feed-forward circuits that form a robust, self-reinforcing signaling network. An example of this is negative auto-regulation of AR transcription (22,23) and reciprocal feedback between AR and PI3K signaling, which results in sensitivity to dual targeting of both pathways (24). Identification of clinically relevant targets that regulate AR function, as well as the key downstream pathways, is critical for more effective treatment of PCa.

## Methods

### Cell Culture

Unless stated otherwise, all cell lines were verified by genetic profiling of polymorphic short tandem repeat (STR) loci as per ATCC standards. We used either AmpFISTR test or GenePrint10 test (Promega, Madison, WI) and analyzed all data using GeneMapper v4.0 software. LNCaP, C4-2, VCaP, PC3, PNT1a, RWPE-1, DUCaP, 22Rυ1, and DU145 cells were obtained from commercial suppliers and grown in RPMI cell culture medium containing 10% fetal bovine serum (FBS) and 1% penicillin/streptomycin in a humidified incubator at 37 ºC with 5% CO_2_.

R1-AD1 was a subline derived from the CWR-R1 cell line. The identity of R1-AD1 was authenticated by positivity for the H874Y point mutation in the AR LBD as determined by polymerase chain reaction (PCR) and Sanger sequencing, and negativity for copy number imbalances along the length of the AR gene was determined by multiple ligation-dependent probe amplification (MLPA) assay. The identity of R1-D567 was authenticated by PCR and Sanger sequencing of the signature break fusion junction generated by transcription activator-like effector endonuclease (TALEN)–based genome engineering.

### Patient Selection and PCa TMA Construction

Prostate tissues were obtained from 359 patients with a median age of 64 years (range = 46–74 years) who underwent radical prostatectomy between 1993 and 2003. Samples and clinico-pathological data were retrieved from the files of the Department of Pathology, Centro Hospitalar do Porto, Portugal, and tissue microarray blocks (TMAs) were prepared. Prior to TMA construction, haematoxylin and eosin (H&E)–stained tumor sections of each radical prostatectomy specimen were re-assessed using the modified Gleason and 2010 pTNM classification (25). Representative areas of adjacent non-neoplastic prostate tissue, PIN lesions, and PCa were selected from the peripheral zone where cancer develops. Three cores from each sample (2mm in diameter, with 0.8mm between core centers) were incorporated into paraffin-blocked TMAs, using a TMA workstation (TMA builder, Beecher Instruments Inc. Technology). Four micron sections were cut for immunohistochemistry (IHC), and an H&E-stained section from each TMA block was reviewed to confirm the presence of morphologically representative areas of the original tissues. The work was approved by the Departamento de Ensino Formação e Investigação (DEFI) Ethics Committee of Centro Hospitalar do Porto (ref. no. 017/08, 010-DEFI/015-CES).

### Generation of Tumor Xenografts

All experiments were carried out in compliance with the UK Animals (Scientific Procedures) Act 1986 under a project licence with the approval of the Cancer Research UK Cambridge Institute Animal Welfare and Ethical Review body. Xenografts were initiated in NOD scid gamma mice (n = 6 per group) by subcutaneous injection of 1 million C4-2b-luciferase (C42b-Sh#1 and nontargeting shLuc C4-2b) cells in 100 µL (50:50 phosphate buffered saline/matrigel, BD Bioscience, San Jose, CA). Mice were given doxycycline in drinking water (0.5mg/mL in 2% sucrose solution) while control mice received 2% sucrose solution. Tumor size was measured with callipers weekly and calculated using the formula volume = (π/6)/abc or (π/6)/abb (if only 2 diameters are available), and a, b, c are the orthogonal axis of the tumor. Tumors were also monitored weekly by luminescence imaging after intraperitoneal injection of 150mg/kg d-luciferin (Caliper Life Sciences) using Xenogen Imaging Analysis software Living Image 3.0 (Caliper Life Sciences, Hopkinton, MA), and luminescence was plotted as photons per second for graphic analysis of growth kinetics. Turbo red fluorescent protein (tRFP) emission was recorded using Xenogen camera once after three weeks while luciferase expression was the measured weekly. Mice were culled at the completion of experiment or when tumors reached 10% of body weight.

### Statistical Analysis

Chi square tests in R were used to look at the association of clinical variables with CHKA, all dichotomized. Survival analysis of the patient data was carried out using the survival package (26). Cox regression analysis of time to biochemical recurrence-free survival was carried out. Proportionality of hazards was confirmed by plotting them. There were several missing values in the dataset, so analysis was done on a complete case basis (cases complete for survival and clinical information). The analysis was initially carried out using CHKA as a predictor value, subsequent models adjusted for each dichotomized clinical variable individually to see what effect it had on the CHKA predictor.

Recursive partitioning (RP) using a conditional inference framework was used to find a significant cutoff for each input gene for predicting recurrence-free survival using a prostate cancer gene expression dataset with accompanying survival data from 79 patients (27). This was implemented using the ctree (28) function in the R (R Core Team, 2014). Kaplan-Meier survival curves and recursive partitioning plots were displayed for those genes that are predictive of recurrence-free survival, with *P* values of less than .05 (corrected for the testing of multiple cutoffs, but not genes). Further details are given in the Supplementary Methods (available online). All statistical tests were two-sided.

### Supplementary Materials

Supplementary information includes experimental procedures; Supplementary Table 4 (available online) contains all the genomic data used in the manuscript including GEO accessions or web links. The probe set used in each data is also specified. The RNA-seq data generated during this work has been submitted to Gene Expression Omnibus and is available for viewing at the following link http://www.ncbi.nlm.nih.gov/geo/query/acc.cgi?token = ytazouoixxedjal&acc=GSE63700.

## Results

### The AR Kinome Suggests CHKA as a Clinically Relevant Target

**Figure 1.**
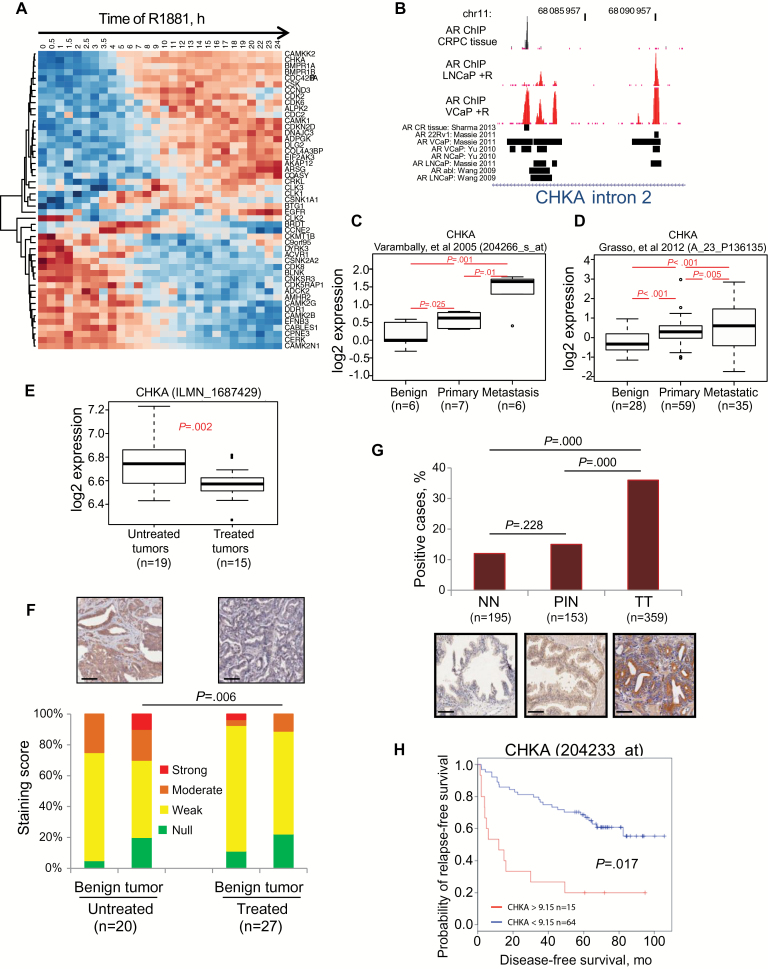
Identification of choline kinase alpha (CHKA) as a clinically relevant androgen receptor (AR) target in prostate cancer (PCa). **A**) Gene expression time course heatmap showing androgen regulation of kinases in LNCaP cells treated with 1nM R1881 androgen. Kinases identified as androgen-regulated on both Illumina beadarrays and SYBR Openarray Human Kinome panel are shown (values represent median centred Illumina beadarray). **Blue color** designates lower expression, and **red** indicates higher expression. **B**) Chromatin immuno-precipitation sequencing analyses from multiple studies on castration-resistant prostate cancer tissue and androgen (R1881)-stimulated PCa cell lines (8, 17, 62, 63) illustrating intron-2 of the genomic locus of the CHKA gene and AR binding sites (coordinates reference GRCh38). **C-D) Boxplots** show CHKA transcript expression in benign tissue, primary PCa, and metastatic PCa in two independent gene expression datasets (30,31). **Boxplots** show interquartile range (IQR), 95% confidence interval (CI), and outlier points. **E) Boxplot** showing CHKA transcript levels in tumors from PCa patients treated with the luteinizing hormone-releasing hormone analogue degarelix, compared with tissue from untreated control PCa patients of matched disease stage. **Boxplots** show IQR, 95% CI, and outlier points. **F**) Immuno-histochemical staining intensity score of CHKA protein expression in the benign adjacent epithelia (in duplicates) and the prostate tumors (in triplicates) of men seven days after treatment or no treatment with degarelix; *P* = .006 two-sided Wilcox rank-sum test of IHC intensity. Representative images inset above. **Scale bar** = 100 µm. **G) Bar plots** showing prevalence of CHKA human staining in non-neoplastic (NN; 195 case patients), prostate intraepithelial neoplasia (PIN; 153 case patients), and tumor tissue (TT; 359 case patients) as a percentage of total case patients. Representative images inset below. **Scale bar** =100 µm. **H**) Kaplan-Meier survival curve from recursive partitioning analysis showing that high CHKA transcript levels are associated with poor recurrence-free survival in the Glinsky cohort (*P* = .017) (27). AR = androgen receptor; CHKA = choline kinase alpha; CI = confidence interval; CRPC = castration-resistant prostate cancer; IQR = interquartile range; LHRH = luteinizing hormone-releasing hormone; R = R1881.

To identify a therapeutically relevant subset of AR targets, we defined the AR-regulated kinome by performing a cross-platform analysis of an androgen-stimulated gene expression time course (17) and a high-throughput real-time PCR kinome array. We identified 49 androgen-regulated kinase genes (Figure 1A), of which 25 were upregulated and 24 were downregulated (Supplementary Table 1, available online). By integrating gene expression with our chromatin immuno-precipitation sequencing (ChIP-seq) datasets (17), we found AR binding sites at the genomic loci of 22 of the 25 androgen upregulated kinases and 8 of the 24 downregulated kinases, providing evidence for direct AR regulation of a subset of these genes (Supplementary Table 1 and Supplementary Figure 1B, available online). Clinical gene expression data revealed that many androgen-upregulated kinases were also upregulated in primary and metastatic PCa (Supplementary Figure 1C and 1D, available online). Importantly, we confirmed the in vivo androgen regulation of a subset of these genes in tumor tissues from chemically castrated PCa patients (n = 15) in contrast to untreated tumors (n = 19) from age/tumor grade-matched patients, finding that 18 of the 25 in vitro androgen-upregulated kinases were statistically downregulated in response to castration in men with PCa (*t* test *P* < .05) (Supplementary Figures 1, E, and 6, A and B, available online).

Among these kinases several have previously been implicated in PCa, including CAMKK2 (17) and IGF-1R (29). Protein interaction network and pathway analysis highlighted clusters of functional enrichment including growth factor receptors, MAPK, TGFβ, and metabolic signaling (Supplementary Figure 1F, available online). These datasets indicated that the *CHKA* gene could be a clinically relevant AR target gene. Supporting this idea, the *CHKA* has strong intragenic AR binding sites in PCa cell lines and in PCa tissue (Figure 1B and Supplementary Figure 2, A and B, available online). CHKA mRNA was statistically significantly overexpressed in localized (primary) and metastatic PCa (30,31) (Figure 1, C and D) and was androgen-regulated in PCa cell lines (Supplementary Figure 2, C-E, available online). Of greater biological relevance, CHKA expression was decreased in tissues from patients treated with the LHRH analogue degarelix, both at the transcript (log fold change from 6.75 to 6.59, *P* = .002) and protein level compared with control patients without androgen deprivation (*P* = .006) (Figure 1, E and F). This, in combination with the known importance of phospho-choline alterations in PCa, prompted us to investigate the importance of CHKA.

CHKA protein expression was frequently increased in PCa (12% positive cases in non-neoplastic [NN] benign prostate compared with 36% positive cases in PCa; *P* < .001) (Figure 1G; Supplementary Figure 2F, available online) and showed some evidence of association with Gleason grade (≤ vs 7) and tumor stage (pT2 vs pT3). There was no evidence of an association of CHKA expression with age, PSA, or biochemical recurrence (Table 1; Supplementary Table 2, available online). However, CHKA was an independent predictor of biochemical recurrence-free survival when other clinical variables were included (Table 2; Supplementary Table 2, and see Supplementary Methods for details, available online), a finding supported by Kaplan-Meier analysis of a separate cohort of clinical samples (CHKA> 9.15, n = 15; CHKA ≤ 9.15, n = 64, *P* = .017) (Figure 1H). Together, these results highlighted that CHKA potentially is a direct AR-regulated gene in vivo whose overexpression is prognostic in PCa.

**Table 1.** χ^2^ square test results for association of dichotomized clinical-pathological data with CHKA (dichotomized, positive/negative)

Parameters	Case patients No. (missing CHKA)	Positive No. (%)	Difference (95% CI)*
Age, y			
≤64	252 (57)	68 (27.0)	1.3% (-9.2 to 11.8)
>64 Missing	222 (59) 006 (5)	59 (26.6) 1 (16.7)	
PSA, ng/mL			
≤5	099 (23)	28 (28.3)	1.7% (-12.7 to 15.3)
>5 Missing	269 (61) 112 (37)	73 (27.1) 27 (24.1)	
Combined Gleason score			
≤7	433 (108)	109 (25.2)	20.3% (-1.6 to 42.2)
>7 Missing	31 (5) 16 (8)	14 (45.1) 5 (31.3)	
Tumor stage			
pT2	359 (92)	86 (24.0)	11.7% (-1.2 to 24.6)
pT3 Missing	98 (16) 23 (13)	36 (36.7) 6 (26.1)	
Biochemical recurrence			
Absent	409 (105)	102 (24.9)	12.7% (-2.7 to 28.2)
Present Missing	69 (15) 2 (1)	25 (36.2) 1 (50.0)	

* Based on nonmissing CHKA values (n = 480). CHKA = choline kinase alpha; CI = confidence interval; PSA = prostate-specific antigen; pT = tumor stage.

**Table 2.** Final model from a backward Cox regression

Variable adjusted for	Comparison	No.* (events)	HR (95% CI)
CHKA	+ve vs –ve	228 (45)	1.88 (1.03 to 3.42)
Tumor stage	pT3 vs pT2	228 (45)	2.18 (1.20 to 3.96)

* The number of case patients is the same to ensure comparability between models and is therefore all the case patients not missing survival and clinical information. The initial model included choline kinase alpha (CHKA), age, prostate-specific antigen (PSA), Gleason stage, and tumor stage. Age was removed first followed by PSA and Gleason, leaving on CHKA and tumor stage in the model. CHKA = choline kinase alpha; CI = confidence interval; pT = tumor stage.

### CHKA is a Eukaryotic Kinase That Acts as a Protein Chaperone

**Figure 2.**
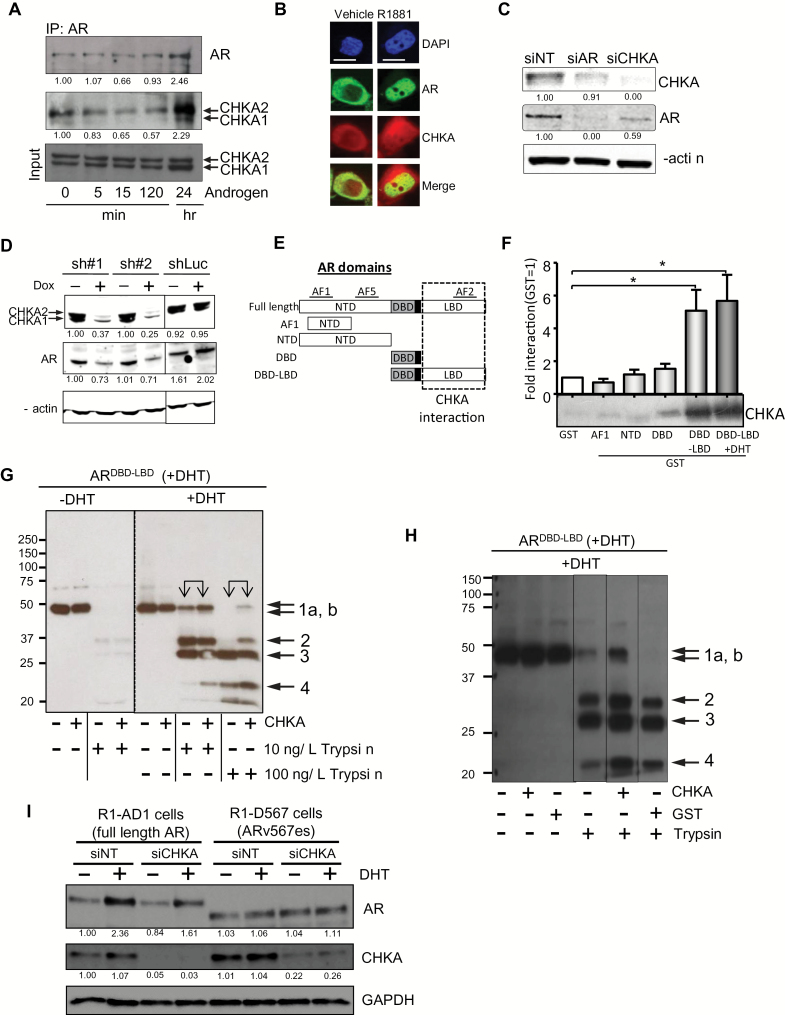
Choline kinase alpha (CHKA) as an androgen receptor (AR) chaperone. **A**) Co-immunoprecipitation showing interaction of endogenous AR and CHKA. LNCaP cell lysates were harvested at the indicated time points and incubated with the AR antibody followed by western blotting using the CHKA antibody. **B**) Fluorescence microscopy images showing intracellular colocalization of CHKA with AR. Images of HeLa cells cotransfected with mCherry-tagged CHKA (**red**) and green fluorescent protein-tagged AR T878A (**green**) and then treated with R1881 (1nM) for two hours. **Scale bar** = 10 µm. **C**) Western blot showing the reduction of expression of CHKA and AR proteins in LNCaP cells transiently transfected with small interfering (si)-RNA targeting either AR (siAR) or CHKA (siCHKA) compared with nontargeting control siRNA (siNT); β-actin is the loading control. **D**) Western blot showing the reduction of expression of CHKA and AR proteins in C4-2b cells expressing two different CHKA small hairpin (sh)-RNAs (sh#1 and sh#2) and shRNA-targeting luciferase (shLuc) in a doxycycline inducible manner; β-actin is the loading control. **E**) Graphic representing several AR truncations employed in the glutathione-S-transferase (GST) pull-down experiment shown in **(F)**. **F**) GST pull-down assay: GST alone and GST-tagged domains of the AR were incubated with CHKA for two hours. CHKA was detected by immunoblotting with an anti-CHKA antibody. The intensity of the immune reactive bands was quantified using Image J and the fold interaction of each domain with CHKA, based on the intensity of the band calculated and plotted relative to GST = 1. Data show mean ± SD, *P < .05 (n = 5). **G**) Protease protection assay of AR^DBD-LBD^. His-tagged AR^DBD-LBD^ was expressed in presence of dihydrotestosterone (DHT) and subjected to proteolytic digestion with 10ng/μL or 100ng/μ: trypsin ± pre-incubation with CHKA (2 picomoles) and ± DHT. Full-length AR polypeptide (band 1a) or proteolytic fragments (bands 1b, 2, 3, and 4) were then detected using an anti-AR C19 antibody. **H**) Protease protection assay of AR^DBD-LBD^. His-tagged AR^DBD-LBD^ was expressed in presence of DHT and subjected to proteolytic digestion with 10ng/μL trypsin ± pre-incubation with CHKA or GST (2 picomoles) and ± DHT. Full-length AR polypeptide (band 1a) or proteolytic fragments (bands 1b, 2, 3, and 4) were then detected using an anti-AR C19 antibody. **I**) Western blot of CHKA and AR in cells engineered to express full-length AR (R1-AD1 cells) and ARv567es (R1-D567 cells). Cells were transiently transfected with nontargeting (siNT) or siRNA targeting CHKA (siCHKA) and treated for 48 hours with vehicle or DHT; GAPDH is the loading control. AF = activation function; AR = androgen receptor; CHKA = choline kinase alpha; DAPI = 4’,6-diamidino-2-phenylindole; DBD = DNA-binding domain; DHT = dihydrotestosterone; GAPDH = glyceraldehyde-3-phosphate dehydrogenase; GST = glutathione-S-transferase; IP = immunoprecipitation; LBD = ligand-binding domain; NTD = amino terminal domain; sh = small hairpin RNA; si = small interfering RNA.

To test whether CHKA influences androgen signaling, we investigated whether CHKA interacts with the AR. Both CHKA isoforms (32) coprecipitated with the AR (Figure 2A). Interestingly, the CHKA2 isoform showed a stronger but transient interaction, which decreased within 5 to 15min of androgen treatment (Figure 2A), consistent with the timing of AR release from cytoplasmic chaperones and its translocation to the nucleus (33). The interaction was regained after 24 hours of androgen treatment, consistent with AR localization returning to a steady state and with androgen-induced expression of CHKA protein at this time point (Supplementary Figure 2C, available online). Evidence of an interaction between the AR and CHKA was also observed in a reciprocal co-immunoprecipitation (CoIP) assay (pulling down CHKA and western blotting for the AR) (Supplementary Figure 2G, available online). Fluorescence microscopy imaging of m-Cherry tagged CHKA (red) and green fluorescent protein tagged AR (GFP-AR; green) revealed evidence of colocalization between CHKA and the AR both in the cytosol and nucleus (Figure 2B; Supplementary Table 2, available online). Surprisingly, AR protein levels were reduced not only by AR knockdown but also by CHKA knockdown using either siRNA (Figure 2C) or a panel of inducible shRNAs (Figure 2D), indicating a reciprocal role of CHKA in regulating AR protein levels.

To determine whether the interaction between the AR and CHKA was direct or indirect, we screened several AR domain constructs (Figure 2E). Using a pull-down assay, we mapped a direct physical interaction between recombinant CHKA2 and the LBD of AR (AR^LBD^) (Figure 2F). Notably, this is the AR domain that interacts with HSPs that stabilize AR (34). We therefore asked whether CHKA could also stabilize AR conformation in protease protection assays using an AR^DBD-LBD^ polypeptide expressed by bacterial cells grown in the presence or absence of androgen dihydrotertosterone (DHT) and with subsequent incubation ± DHT with a recombinant CHKA2. In the presence of both DHT and CHKA protein, there was reduced cleavage of the AR^DBD-LBD^ polypeptide at both low and high concentrations of trypsin (Figure 2G, arrows 1a & 1b), as well as increased resistance to cleavage of 21 and 35kDa fragments, particularly at higher trypsin concentrations (Figure 2G, arrows 2 & 4). Control experiments using glutathione-S-transferase (GST) suggested a specific protection of the full-length AR^DBD-LBD^ in the presence of CHKA but not GST (Figure 2H, arrows 1a & 1b). Similarly, there was a reduced cleavage of AR polypeptide fragments in the presence of CHKA (Figure 2H, arrows 2 & 4). We found that AR^DBD-LBD^ expressed in the absence of DHT was extremely sensitive to complete trypsin digestion (Supplementary Figure 2H, available online) and this could be reversed by the addition of both DHT and CHKA, although not by DHT or CHKA individually. Together these data suggest that CHKA exerts a chaperone function on the AR.

We tested whether CHKA binding stabilized AR via binding to the AR^LBD^ using R1-D567 cells that exclusively express an AR variant (ARv567es) lacking the LBD (10). This variant is constitutively active and promotes prostate carcinogenesis (35), and its expression is associated with resistance to enzalutamide and abiraterone (11). While CHKA knockdown did not alter ARv567es expression in R1-D567 cells (Figure 2I), it decreased expression of DHT-stabilized full-length AR in isogenic R1-AD1 cells. These findings validate biochemical data and support a model in PCa whereby a chaperone function of CHKA stabilizes the AR exclusively via binding to the AR^LBD^.

### Reliance of the AR Transcriptional Program on CHKA in PCa

**Figure 3.**
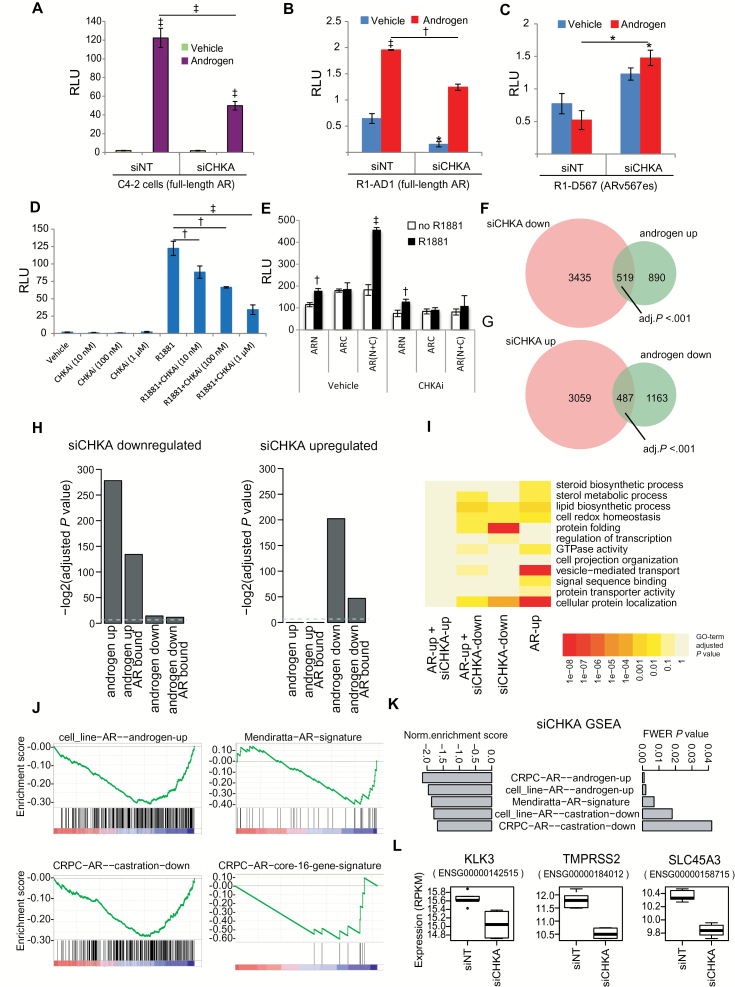
Effect of choline kinase alpha (CHKA) on the androgen receptor (AR) transcription program. **A**) Luciferase reporter assay showing AR transactivation potential in C4-2 cells transiently transfected with MMTV-Luc and treated ± siCHKA and ± androgen (1nM R1881) for 48 hours; **bars** show mean ± SD (n = 3). *P* value by two-sided Student’s *t* test. **B-C**) Luciferase reporter assay showing AR transactivation potential in R1-D567 and R1-AD1 cells transfected with PB3-Luc and treated ± siCHKA ± androgen (DHT, 10nM); **bars** show mean ± SD (n = 3). **D**) Luciferase reporter assay of C4-2 cells transiently transfected with MMTV-Luc showing the dose response to CHKAi in cells treated with R1881 as indicated; **bars** show mean ± SD (n = 3). **E**) Bipartite interdomain N-C interaction based reporter assay in C4-2 cells. Cells were transiently transfected with MMTV-Luc ± pSVARN (ARN, AR N-terminus) and/or ± pSVARC1 (ARC, AR C-terminus) for 48 hours ± R1881 (1nM) and ± CHKAi (1 μM); **bars** show ± SD (n = 3). **F) Venn diagram** showing the overlap between siCHKA-downregulated genes and androgen-upregulated genes and **(G) Venn diagram** showing the overlap between siCHKA-upregulated genes and androgen-downregulated genes. *P* values are Bonferroni-corrected hypergeometric tests (see below for details). Genes differentially expressed following CHKA siRNA knockdown were selected from biological triplicate experiments using a false discovery rate cutoff of .05. Androgen-regulated genes were selected from an androgen treatment time course experiment in LNCaP cells, using autocorrelation values above background simulations to identify regulated genes. A core set of direct androgen-regulated genes was defined by taking such genes with AR binding site(s) within 25kb. **H) Barplots** of -log2 transformed *P* values comparing overlaps of up- and downregulated gene sets from siCHKA and AR-stimulated conditions. To assess the significance of the overlap between siCHKA and AR stimulation expression changes, we applied hypergeometric tests and resultant *P* values were adjusted for multiple testing using the Bonferroni correction. **I**) Heatmap summary of functional annotations for overlapping AR- and CHKA-regulated genes: Enrichment of functional annotations for each gene set was calculated using the DAVID Gene Ontology tool, and Benjamini-adjusted *P* values are plotted. **J**) Gene set enrichment analysis (GSEA) plots of CHKA siRNA-ranked gene expression changes, highlighting AR-bound genes in cultured cell lines or in CRPC tumor tissue, which was regulated by androgen treatment in cultured cells or by castration in xenografts. **K) Barplot** summary of GSEA-normalized enrichment scores and FWER corrected *P* values for the CHKA siRNA gene expression profile compared with AR gene sets. Gene sets shown in **(J-K)** were from Sharma et al. (2013) and Mendiratta et al. (2009). **L) Boxplots** showing the expression values for three established AR-regulated genes following CHKA siRNA (siCHKA) transfection. Data show results of six biological replicates; **boxplots** show interquartile range, 95% confidence interval, and outlier points. * = *P* < .5; † = *P* < .01; ‡ = *P* < .001. CHKA = choline kinase alpha; CHKAi = choline kinase alpha inhibitor; FWER = family-wise error rate; CRPC = castration-resistant prostate cancer; GSEA = gene set enrichment analysis; MMTV = mouse mammary tumor virus; NT = nontargeting; RLU = relative light units; si = small interfering RNA.

To further investigate the functional consequences of CHKA on AR signaling, we used a reporter assay to assess AR transactivation capacity in C4-2 PCa cells and observed a 2.5-fold decrease (*P* < .001) in R1881-induced AR transcriptional activity following CHKA knockdown using siCHKA (Figure 3A). CHKA knockdown decreased full-length AR transcriptional activity in R1-AD1 cells by 2-fold (*P* < .005) (Figure 3B) but failed to inhibit ARv567es variant (10) lacking the LBD in R1-D567 cells (Figure 3C), indicating that CHKA regulates AR transcriptional activity by binding to AR^LBD^, consistent with our biochemical data. As an additional approach to determine the role of CHKA in regulating AR signaling, we used hexadecyl trimethylammonium bromide (referred to as CHKAi), a structural analogue of choline that binds to CHKA and inhibits conversion of choline into phosphocholine (36), a metabolic reaction in the Kennedy pathway that is catalyzed by CHKA. Like CHKA knockdown, CHKAi decreased androgen-induced AR transcriptional activity in a dose-dependent manner from 122-fold (R1881 alone) to 34-fold (*P* < .001; R1881 with 1 µM CHKAi) in C4-2 cells (Figure 3D). The level of inhibition was equivalent to that of the new-generation AR antagonist (enzalutamide) and statistically significantly greater than a first-generation AR antagonist bicalutamide (Supplementary Figure 3A, available online). Using a bipartite N-C AR reporter assay (37), we found that CHKAi abrogated the AR N-C interdomain interaction that is associated with agonist-induced transcriptional activity and stability of the AR, consistent with a chaperone function of CHKA (Figure 3E). In line with this, CHKAi repressed CHKA and AR protein levels to a similar extent with CHKA knockdown (Supplementary Figure 3B, available online). CHKAi also decreased androgen-stimulated nuclear AR levels (Supplementary Figure. 3C, available online).

Transcriptome sequencing from CHKA knocked down C4-2 cells highlighted the global impact of CHKA on the AR transcriptome. We found a statistically significant bidirectional overlap between siCHKA and androgen-regulated genes (519 genes, 58% of the androgen-upregulated transcriptome; 487 genes 42% of the androgen-downregulated genes, hypergeometric *P* < .001) (Figure 3, F and G). Among the siCHKA-downregulated genes, almost 50% showed evidence of direct AR regulation (AR binding within 25kb) (Figure 3H), which is consistent with a global role for CHKA in AR-dependent transcriptional regulation. Pathway analysis identified common cellular processes regulated by the AR and CHKA, including enrichment of sterol metabolic processes, lipid biosynthetic processes, cell redox homeostasis, and GTPase activity (Figure 3I). Most notably, the CHKA knockdown signature was enriched for pathways regulating protein folding and cellular protein localization, consistent with its chaperone function identified herein. An orthologous gene set enrichment analysis following CHKA knockdown revealed a statistically significant enrichment of AR-bound, androgen-regulated genes identified in multiple datasets from cell lines and castration-resistant prostate cancer (CRPC) (FWER *P* < .05) (Figure 3, J and K). We confirmed that archetypal AR target genes including *KLK3* (PSA), *TMPRSS2*, and *SLC45A3* were downregulated by CHKA knockdown (Figure 3L) and that the effects of CHKA depletion on endogenous AR targets only occurred in androgen-stimulated conditions, indicating that these effects are AR dependent (Supplementary Figure 3D, available online). These analyses highlight a functional link between AR and CHKA signaling, consistent with our biochemical and reporter gene studies, indicating a cooperative function of CHKA in driving the AR-regulated transcriptome by stabilizing AR protein levels.

### Effect of CHKA Inhibition on PCa Growth

**Figure 4.**
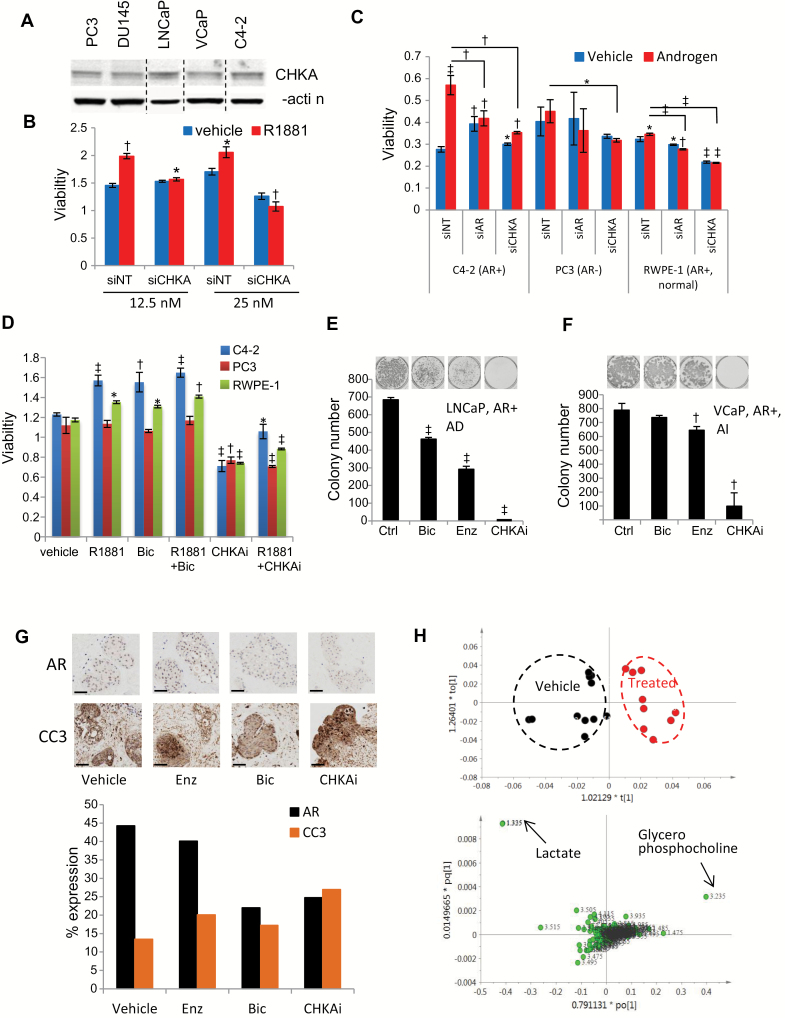
Effect of choline kinase alpha (CHKA) inhibition on prostate cancer (PCa) growth. **A**) Western blot showing CHKA expression in five PCa cell lines; β-actin is the loading control. **B**) MTS assay of LNCaP cells transfected with two concentrations of siNT or siCHKA. Cells were grown in the presence or absence of 1nM R1881 for 120 hours; **bars** show mean ± SD (n = 3). *P* values by two-sided Student’s *t* test. **C**) MTS assay in three transiently transfected PCa cell lines with 25nM siNT, siCHKA, or si-androgen receptor (AR) in response to androgen R1881 (1nM); **bars** show mean ± SD (n = 3). *P* values by two-sided Student’s *t* test. **D**) MTS viability assay showing cell viability in PCa cells treated with R1881 (1nM), bicalutamide (1 µM), and CHKAi (1 µM) data show mean ± SD (n = 3). *P* values by two-sided Student’s *t* test. **E-F**) Clonogenic cell survival assay in LNCaP **(E)** and VCaP **(F)** cell lines treated for 14 days with bicalutamide, enzalutamide, or CHKAi (all at 10 µM); **bars** show mean ± SD (n = 3). *P* values by two-sided Student’s *t* test. **G**) **Upper panel**; **photo micrographs** of PCa explants treated with the drugs indicated. **Scale bar** = 100 µm. **Lower panel**; **barplot** showing the percentage of nuclei positive for the expression of AR and CC3 in human PCa tissue cultured ex vivo and treated with enzalutamide, bicalutamide, or CHKAi (10 µM). **H) Upper panel**: **scores plot** from principal component analysis of ^1^H NMR metabolite profiles of LNCaP cells. Control (**black dots**) and CHKAi treated (**red dots**). **Lower panel** shows loadings plot from principal component analysis of ^1^H NMR metabolite profiles. * = *P* < .05; † = *P* < .01; ‡ = *P* < .001. AD = androgen-dependent; AI = androgen-independent; AR = androgen receptor; Bic = bicalutamide; CC3 = cleaved caspase 3; CHKA = choline kinase alpha; CHKAi = choline kinase alpha inhibitor; Ctrl = control; Enz = enzalutamide; nM = nanomolar; NMR = nuclear magnetic resonance; NT = nontargeting; Si = small interfering RNA.

Because of its vital role in the Kennedy pathway, all investigated PCa cell lines express CHKA (Figure 4A), providing an opportunity to interrogate the effects of CHKA inhibition on androgen-induced cell growth. CHKA inhibition by siRNA-mediated knockdown and also by CHKAi treatment effectively decreased the confluence of C4-2 cells in a manner similar to AR inhibition (Supplementary Figure 4A, available online). CHKA knockdown in LNCaP cells led to a dose-dependent reduction in androgen-stimulated growth in these AR-expressing cells (1.3- and 2.0-fold reduction, with 12.5nM and 25nM siCHKA, respectively) (Figure 4B). In a panel of AR-positive PCa cells, CHKA knockdown caused inhibition of androgen-stimulated growth comparable with the effects of AR knockdown but had only a modest effect on the growth of AR-null PCa cells PC3 and DU145 PCa cells and normal prostate epithelial PNT1a and RWPE-1 cells (Figure 4C; Supplementary Figure 4, B and C, available online). Similarly, CHKAi had profound growth inhibitory effects on AR-positive C4-2 and LNCaP-LN3 cells, which were partially reversed by the synthetic androgen R1881. CHKAi only modestly inhibited growth of PC3, DU145, and PNT1a cells (Figure 4D; Supplementary Figure 4, D and E, available online). These data are collectively consistent with a greater effect of CHKA on the growth of AR-expressing PCa cells, likely resulting from dual inhibition of AR signaling and the Kennedy pathway in these cells. In clonogenic survival assay with LNCaP cells, AR antagonists bicalutamide and enzalutamide decreased colony number by 1.5- (*P* < .001) and 2.3-fold (*P* < .001), respectively, while CHKAi reduced the clonogenic survival of androgen-dependent LNCaP by more than 50-fold (*P* < .001) (Figure 4E). Similar results were obtained for another androgen-dependent cell line DUCaP (Supplementary Figure 4F, available online). In androgen-independent VCaP cells, bicalutamide was not effective at clonal repression while enzalutamide was marginally effective (1.3-fold, *P* < .01); however, CHKAi treatment led to a 7-fold decrease in clonogenic potential (*P* < .01) (Figure 4E). Results with similar trends were obtained for androgen-independent LNCaP-LN3 (Supplementary Figure 4G, available online). The growth-promoting role of CHKA does not depend on the catalytic activity of CHKA because the addition of exogenous phosphocholine or phosphatidylcholine did not rescue growth inhibition by CHKAi (Supplementary Figure 4H, available online). In an ex vivo culture assay, treatment of hormone-naïve primary PCa tissue with bicalutamide and CHKAi decreased AR expression in tumor epithelia while both anti-androgens and CHKAi increased levels of cleaved caspase-3, a marker of apoptosis (Figure 4G). Concordantly, CHKAi treatment increased the content of glycerophosphocholine (Figure 4H), a metabolic marker of apoptosis. CHKAi also increased apoptosis, and this increase was reversed by androgen co-treatment (Supplementary Figure 4I, available online). Collectively, these experiments indicate that CHKA inhibition opposes androgen action, thus triggering apoptosis in PCa cells and tissue.

### Impact of CHKA Knockdown on Tumor Growth and Invasion

**Figure 5.**
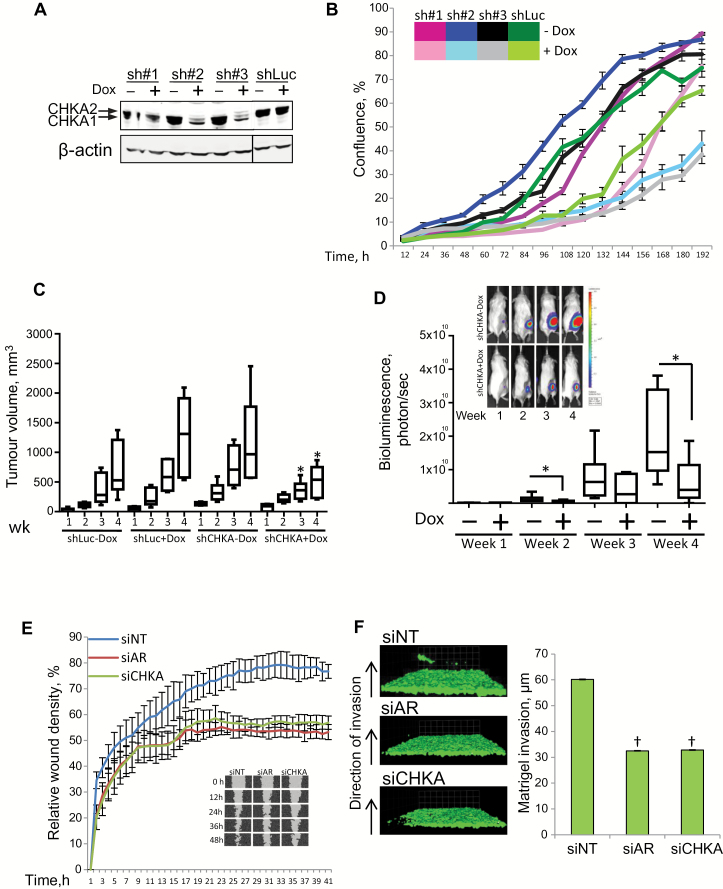
Effect of choline kinase alpha (CHKA) depletion on metastatic features and tumor progression. **A**) Western blot showing expression of CHKA in clones of C4-2b cells with dox-inducible small hairpin (sh)-RNA targeting CHKA (sh#1, sh#2, sh#3, or shLuc control) treated with doxycycline (1 μM) for 72 hours; β-actin is the loading control. **B**) Growth profiles for C4-2b cell clones (sh#1, sh#2, sh#3, and shLuc control) in response to doxycycline induction of the respective shRNAs; **lines** show mean (n = 6). **Error bars** represent SD. **C-D) Boxplot** showing progression of tumor xenografts of shLuc and shCHKA C4-2b cells measured over four weeks (n = 6 per group) by **(C)** callipers to determine the volume and **(D)** by Xenogen camera to measure bioluminescence. Significance was calculated by the Mann-Whitney U test.

To understand the effect of perturbing CHKA function on tumor growth kinetics, we used the highly aggressive C4-2b luciferase cell line and engineered it to express three distinct CHKA shRNAs (sh#1, sh#2, and sh#3) in a doxycycline-inducible manner. In the presence of doxycycline, sh#1 did not robustly decrease CHKA levels, but the two other CHKA shRNAs (sh#2 & sh#3) were effective at decreasing CHKA levels (Figure 5A). These shRNAs particularly reduced levels of the CHKA2 isoform that had shown stronger interaction with AR (Figures 2A and 5A). Consistent with the decreased CHKA levels in cells expressing sh#2 and sh#3, we observed a reduced growth rate in both of these shRNA clones following doxycycline induction compared with either sh#1 or shLuc cells (Figure 5B). Xenograft tumors were generated with CHKA sh#2 cells, which upon treatment with doxycycline grew slower than shCHKA xenograft without doxycycline treatment (week 3 and week 4, Mann-Whitney *P* < .05) (Figure 5C) and showed a reduced bioluminescent growth profile than in tumors in the nondoxycycline control group (week 4, Mann-Whitney *P* < .05) (Figure 5D). The doxycycline-mediated induction of shRNA was confirmed by the induction of turbo red fluorescent protein (Supplementary Figure 5A, available online). Further analysis of the excised tumor xenografts confirmed a tendency towards decreased CHKA levels and clearly decreased AR protein expression in CHKA sh#2 xenografts from mice treated with doxycycline (Supplementary Figure 5, B and C, available online). CHKA activity can be considered to have conferred an invasive phenotype on PCa cells because both CHKA and AR depletion delayed “wound healing” in a scratch wound assay to a comparable extent (1.6-fold decrease as compared with siNT) (Figure 5E) and similarly decreased invasion in both a Matrigel invasion assay (*P* < .001) (Figure 5F) and a Boyden Chamber assay (Supplementary Figure 5D, available online). These observations are consistent with PCa cells requiring CHKA and the AR for motility, as suggested by transcriptome analysis enrichment of pathways regulating GTPase activity (Figure 3I).

**Boxplots** show data spread, **boxes** show interquartile range, **error bars** show 95% confidence intervals, and **data points** show outlier values. **E**) Scratch wound assay with VCaP cells showing the time course of wound closure after transient transfection with the small interfering (si)-NT, si-androgen receptor (AR), or siCHKA; **lines** show ± SD (n = 4). **F**) Matrigel-based inverted invasion assay with GFP expressing C4-2 cells transiently transfected with the siRNAs shown; **bars** show means ± SD (n = 3), significance calculated by D’Agostino & Pearson omnibus normality test, *P* < .0001. * = *P* < .05; † = *P* < .0001. AR = androgen receptor; CHKA = choline kinase alpha; Dox = doxycycline; NT = nontargeting; sh = small hairpin; si = small interfering RNA.

The data presented here demonstrate the importance of CHKA inhibition in decreasing AR levels and repressing PCa growth and invasion, highlighting the potential future benefits of inhibitors of this kinase in the clinical management of PCa.

## Discussion

By employing genome-wide approaches, we characterize the AR-regulated kinome in PCa and provide direct evidence that CHKA is a chaperone for AR, promoting its stability and function. Indeed, to our knowledge this is the first report indicating that kinases can act as chaperones. Decreasing CHKA protein levels, both by siRNA knockdown and CHKA inhibitor, antagonized AR signaling and constrained development of aggressive phenotypes in models of PCa. We propose that CHKA promotes AR signaling and that therapeutic targeting of CHKA has the potential to improve management of advanced PCa.

**Figure 6.**
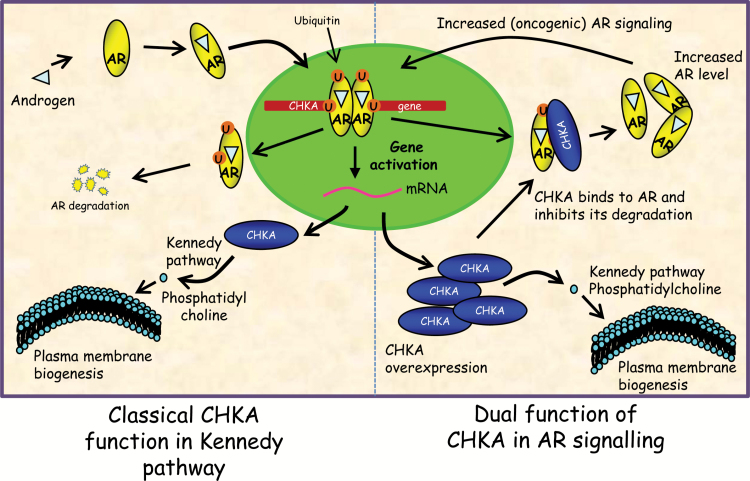
Proposed model depicting classical and nonclassical functions of choline kinase alpha (CHKA). Classical model involves ubiquitination and activation of androgen receptor (AR)–dependent transcription, which results in upregulation of CHKA, which is required for the execution of the Kennedy pathway to produce phosphatidylcholine required for plasma membrane biogenesis. In addition to its role in Kennedy pathway, CHKA can also interact with the AR in cytoplasm and promotes its stability (nonclassical). This could lead to AR overexpression and increased AR signalling, which in turn may allow for more CHKA production. AR = androgen receptor; CHKA = choline kinase alpha; mRNA = Messenger RNA.

Initial profiling of the AR-regulated kinome in PCa revealed functionally and clinically important signaling events downstream of the AR in PCa. Within the AR-regulated kinome, we characterized CHKA as a chaperone that regulates AR signaling, elucidating a feed-forward AR-CHKA signaling loop that reinforces AR activity and allows CHKA to maintain its expression in PCa. We show that the chaperone function of CHKA confers a growth advantage on PCa by stabilizing the AR in addition to its well-known function in fuelling membrane production through the Kennedy pathway (38) (Figure 6), suggesting a de facto role for CHKA as a rate-limiting factor in cancer cell growth.

In advanced PCa models, AR knockdown is very effective in repressing tumor growth (39,40), but this has not yet been achieved long-term in the clinic. Recently, targeting the expression and/or activity of AR and its interacting networks using enzalutamide and abiraterone has established the paradigm of inhibiting the AR axis for the treatment of CRPC (4,5), providing impetus for the development of more effective inhibitors to target the AR axis (41,42). Unfortunately, resistance to these new therapies targeting AR signaling has begun to emerge, primarily involving amplification or gain-of-function point mutations and potentially splice variants in the AR gene (7,11). This indicates strong dependence of advanced PCa on continued AR signaling and provides a unique opportunity to target other facets of AR full-length signaling, including chaperones such as CHKA, to decrease AR levels.

The discovery that CHKA acts as a chaperone provides a potential explanation for an earlier study in breast cancer, which reported promotion of cancer cell growth by both wild-type and kinase-dead CHKA mutants, suggesting that the CHKA protein level per se, and not its catalytic function, is required for tumor cell growth (43). Another recent report on breast cancer cell growth comparing CHKA knockdown with catalytic inhibition by CHKA inhibitors also suggested that downregulation of CHKA protein levels, and not merely the catalytic activity, is required for cancer cell survival (44), further supporting the concept that CHKA may have a dual chaperone and kinase function in cancer. Indeed, independent of their catalytic function, kinases can regulate diverse cellular processes, including stimulation of DNA synthesis (45), allosteric effects on enzymes promoting cell survival (46), and the scaffolding of protein complexes (47). Our work therefore provides a conceptual advance in kinase biology by showing that a kinase can act as a chaperone, in this case for the AR, which may provide one explanation for the emergence of resistance to kinase inhibitors in a range of tumors where only the catalytic function of a kinase is targeted (48–50).

A number of studies have documented the overexpression of CHKA protein in multiple cancer types including breast, colorectal, endometrial, lung, ovarian, and prostate (51,52). Consistent with this, many solid tumors, including PCa, exhibit an increased phosphatidylcholine:choline ratio in tumor foci (53). Therefore, CHKA is being pursued as a therapeutic target and prognostic marker in advanced disease for a range of tumor types (54–56). Indeed, a phase 1 study of a CHKA inhibitor for the treatment of solid tumors has recently completed recruitment (ClinicalTrial.gov identifier NCT01215864). Our work identifies a specific clinical niche for future phase I and II trials in PCa using CHKA inhibitors. This discovery also enhances the prospects for personalized PCa therapy by stratifying patient cohorts according to their AR status. Majority of PCa express a full-length AR mutated in the LBD and/or have amplification of AR contributing to resistance to conventional ADT. These patients are more likely to benefit from therapeutic targeting of CHKA than the subset of patients expressing only AR variants such as AR-V7, which lacks the AR^LBD^ and would be predicted to not interact with CHKA. Appropriate patient selection can avoid unnecessary overtreatment and toxicity.

CHKA contains multiple “NR” box or LxxLL motifs often found in coregulatory proteins of nuclear receptors. Because the LBD is highly conserved among members of the nuclear receptor superfamily, it is likely that CHKA may function as a chaperone for other nuclear receptors. In view of the approximately 76% sequence homology between the LBD of the glucocorticoid receptor (GR) and AR (57), and the antagonism of the GR by the AR antagonist cyproterone acetate (58), it is plausible that the chaperone function of CHKA may also stabilize GR and other members of the nuclear receptor superfamily. Thus, CHKA inhibition could be an effective therapeutic approach in the treatment of PCa, where AR function is bypassed by GR (59). Our study also provides a rationale for testing a CHKA inhibitor in 60% to 70% cases of molecular apocrine-type breast cancers that rely on AR signaling (60,61).

This study also had some limitations. Firstly, the CHKAi employed in this study is a generic inhibitor and some of its effects on the cell growth may have been more pronounced as compared with the siRNA studies owing to the inhibition of other related kinases, although many experiments with the inhibitor and siRNA produced concordant results. This underscores the need for developing highly specific CHKA inhibitors. Secondly, as CHKA protects AR by binding to its LBD, PCa patients whose tumors are driven by ARVs may still maintain AR signaling despite therapeutic targeting of CHKA. Moreover, the results we reported are underpinned by appropriate statistics, although in some of the functional studies the outcomes should be interpreted against the number of experiments and variance we reported.

The discovery of a chaperone function of CHKA for AR signaling might also have wider implications in the treatment of other cancers and should accelerate the screening of potential CHKA inhibitors that will not only inhibit the catalytic function but also decrease the CHKA protein level. We believe our work will direct efforts in the treatment of PCa in particular, but also for other cancers, by stimulating a systematic re-evaluation of CHKA as a therapeutic target, especially in tumors where it is overexpressed and may exhibit chaperone-analogous effects on other oncogenic proteins.

## Funding

This work was supported by a Cancer Research UK program grant (to DEN) and also by the US Department of Defense (Prostate Cancer Research Program Transformative Impact Award, grant ID W81XWH-13-2-0093 to WDT and SMD), PCFA/Cancer Australia/Movember (grant IDs 1012337 and 1043482 to WDT and LAS), Cancer Australia (grant ID 1043497 to WDT and JC), and The Ray and Shirl Norman Cancer Research Trust (to WDT and LAS). The Dame Roma Mitchell Cancer Research Laboratories were supported by an establishment grant from the Prostate Cancer Foundation Australia (ID 2011/0452). FO was supported by a PhD project grant from Prostate Cancer UK (S10-10). LAS is supported by a Young Investigator Award from the Prostate Cancer Foundation (the Foundation 14 award).

## Supplementary Material

Supplementary Data

## Notes

Study sponsors had no role in the design of this study; the collection, analysis, or interpretation of the data; the writing of the manuscript; or the decision to submit the manuscript for publication. We acknowledge members of the Uro-Oncology laboratory for helpful discussions. We thank research staff of Cancer Research UK Cambridge Institute core facilities for technical support. We are very grateful to Dr. Davina Honess for critically reading the manuscript. We acknowledge Dr. Curtis Pettaway for the gift of LNCaP-LN3 cells and Dr. Zaver Bhujwalla and Dr. Tariq Shah for CHKA cDNA. We thank the National Institute for Health Research, Hutchison Whampoa Limited, the Human Research Tissue bank (Addenbrooke’s hospital Cambridge), and Cancer Research UK. The authors would like to thank those men with prostate cancer who have donated their time and samples to the Cambridge Biorepository, which were used in this research. We also would like to acknowledge the support of the research staff in S4 who so carefully curated the samples and the follow-up data (Jo Burge, Marie Corcoran, Anne George, and Sara Stern).
