# Supplementary material for: Choline Kinase Alpha as an Androgen Receptor Chaperone and Prostate Cancer Therapeutic Target
Source: J Natl Cancer Inst. 2015 Dec 11;108(5):djv371. doi: 10.1093/jnci/djv371 (PMC4849803; doi:10.1093/jnci/djv371)
Supplement: Supplementary Data [file supp_108_5_djv371__index.html]

Choline Kinase Alpha as an Androgen Receptor Chaperone and Prostate Cancer Therapeutic Target — Supplementary Data 

# Choline Kinase Alpha as an Androgen Receptor Chaperone and Prostate Cancer Therapeutic Target

## Supplementary Data

Data files

- Supplementary Data - Supplementary Data
