# Supplementary material for: Choline Kinase Alpha as an Androgen Receptor Chaperone and Prostate Cancer Therapeutic Target
Source: J Natl Cancer Inst. 2015 Dec 11;108(5):djv371. doi: 10.1093/jnci/djv371 (PMC4849803; doi:10.1093/jnci/djv371)
Supplement: Supplementary Data [file supp_djv371_Supp_mtls_ed.docx]

**SUPPLEMENTARY MATERIALS**

**SUPPLEMENTARY METHODS**

**Reagents/consumables**

Methyltrienolone (R1881) and Enzalutamide were obtained from Perkin Elmer and Axon Medchem respectively. Dihydrotertosterone (DHT), Dimethyl sulfoxide (DMSO), the CHKA inhibitor Hexadecyltrimethylammonium bromide (CHKAi) and bicalutamide were obtained from Sigma chemicals (Dorset, UK). Cell culture medium, foetal bovine serum (FBS) and other cell culture reagents including antibiotics were obtained from Life Technologies (Carlsbad, CA).

**Plasmids, siRNA and Transient transfections**

To achieve potent reduction in target mRNA expression, siRNA ON-TARGETplus smart pools (Dharmacon/Life technologies) were used to knockdown AR and CHKA. Transient transfections with siRNA and plasmids were performed using Lipofectamine RNAiMAX and Lipofectamine2000 transfection reagent respectively (Life Technologies) as per manufacturer’s recommendations. We performed reverse transfections and used 25 nM siRNA in all knockdown experiments except RNA sequencing where 50 nM siRNA was used to attain a near complete depletion of endogenous CHKA. MMTV-Luc and PB3-luc reporter plasmids have described previously ([1](#_ENREF_1), [2](#_ENREF_2)). To construct the mCherry-CHKA expression vector, human CHKA cDNA (Courtesy Dr. Zaver Bhujwalla) was cloned in-frame into pmCherry-C1 vector using the blunt-end ligation method. Protein expression of the mCherry-tagged CHKA was confirmed by Western blotting using a specific antibody against CHKA (data not shown).

**Reporter assays**

In all luciferase assays, Renilla luciferase plasmid (Promega) was used as an internal control. All cells treated with androgens, R1881 or DHT were grown in medium containing hormone-depleted (charcoal-stripped) FBS. Cells were harvested 48 hr post-transfection using the passive lysis buffer provided with the dual luciferase assay reagents (Promega) to measure both luciferase activity and Renilla luciferase activity.

To carry out the bipartite inter-domain interaction (N-C) assay ([3](#_ENREF_3)), PC3 cells were transfected with MMTV-Luciferase reporter, Renilla luciferase reporter, pSAVARN1 (ARN) and/or pSVARC (ARC) ([4](#_ENREF_4)) using lipofectamine2000. At 7 or 8 hr post-transfection the medium was changed for charcoal-stripped serum containing medium and cells were treated with CHKAi (1 µM) and/or R1881 (1 nM). Thirty-six hrs later cells were harvested using the dual luciferase assay reagents and luciferase activity was measured using a luminometer (Pherastar, BMG LABTECH, Ortenberg, Germany). For luciferase assays with R1-AD1/R1-D567, cells were treated with DHT for 20 hr before harvesting. All experiments were carried out in triplicate unless otherwise stated.

**Cell viability assay**

Cells were trypsinised and counted using a Vicell instrument (Beckman Coulter, Brea, CA). Cell growth assays were carried out in 96 well plates (2000-3000 cells per well). Cells were plated and simultaneously treated with the indicated chemicals/drugs and incubated until control vehicle treated cells reached 96-98% confluence (5-7 days for different cell lines). Cell viability was determined by incubation with the MTS reagent (3-(4,5-dimethylthiazol-2-yl)-5-(3-carboxymethoxyphenyl)-2-(4-sulfophenyl)-2H-tetrazolium), followed by colorimetric assay as per manufacturers protocol (Promega, Madison, WI).

**Clonogenic cell survival assay**

Cells were seeded in a 6-well culture dish (Corning Life Sciences, Corning, NY); (1000 cells per well except for LNCaP, where 5000 cells were seeded) and 48 hr later cells were treated with 10 µM of the antagonist/inhibitor. Medium containing drugs was replenished twice a week. Two weeks later, cells were fixed in acetone:methanol (1:1) for 5 min and stained for 10-15 min with Giemsa (from Raymond A Lamb ltd. UK) which was filtered and diluted 1:10 in water). Plates were washed with tap water, air dried and the colonies were counted using a colony analyser (Oxford Optronix, UK). All colonies were counted in every plate except in LNCaP where colonies > 500µm were counted. All experiments were conducted in triplicate.

**Live cell imaging/confluence analyses**

Confluence analysis was performed using the Incucyte instrument (Essen Bioscience Germany). Cells were plated and simultaneously treated with drugs in TPP 96-well culture dishes and placed in the instrument. Experiments were conducted with 8 replicates, recording live cell images every 3 hr.

**Expression and purification of AR domains**

The pGEX-2TK (GST) plasmid and the pGEX-2TK plasmid with AR^AF1^, AR^NTD^, AR^DBD-LBD^ domains were transformed into BL21 (DE3) competent cells (Agilent technologies, Palo Alto, CA). Colonies expressing proteins of interest were seeded in 20 ml LB starter culture and grown overnight at 37 ºC with shaking. Cultures were diluted 1:10 and grown on at 37 ºC with shaking until they reached an optical density of 0.6-0.8. pGEX-2TK-NTD and pGEX-2TK-DBD-LBD were grown in 2X TY medium (1.6% w/v tryptone; 1% w/v yeast extract; 0.5% w/v NaCl). pGEX-2TK, pGEX-2TK-AF1 and pGEX-2TK-NTD were induced with 0.1 mM IPTG for 90 min but pGEX-2TK-DBD-LBD was induced with 0.5 mM IPTG with the addition of 50 μM DHT for 3 hr. All cultures were incubated at 25 ºC with shaking following induction.

Cells were harvested by centrifugation (5500 rpm, 4 ºC for 20 min) and pellets were resuspended in 5 ml buffer A (20 mM Tris-HCl pH 8.0; 50 mM NaCl; 1 mM EDTA; 1 mM dithiothreitol (DTT), 1 mM phenylmethanesulfonyl fluoride (PMSF) and frozen. For pGEX-2TK-DBD-LBD, 50 μM DHT was added. Subsequent following procedures were carried out at 4 ºC.

Frozen cells were thawed on ice and diluted to 10 ml with buffer A (For pGEX-2TK-DBD-LBD, 50 μM DHT was added). 0.5 mg/ml lysozyme was used to lyse cells for 20 min on a roller after which 10 mM MgCl_2_, 1 mM MnCl_2_, 10 μg/ml DNase I and 10 μg/ml RNaseA were added to each lysate before a further incubated for 10 min on the roller. Insoluble cell debris was pelleted by centrifugation at 10,000 rpm for 20 min. the supernatant was kept on ice until purified.

1250 μl glutathione sepharose 4B slurry (GE Healthcare, Piscataway, NJ) was centrifuged to remove ethanol and the pellets were washed with 10 ml cold PBS and re-centrifuged. The supernatant was added to the beads and incubated for 90 min on a roller. The slurry was centrifuged and the supernatant discarded. The beads were washed three times with 10 ml of PBS. For pGEX-2TK-DBD-LBD, 50 μM DHT was added to the PBS and the beads were kept on ice until used. SDS-PAGE was used to analyse protein and the concentration was determined by running BSA standards and beaded-proteins on the same gel.

pET-DBD-LBD (His-tagged) plasmid was expressed with or without DHT as described above, however it was purified using Ni2+ -nitriloacetate agarose affinity chromatography, and dialyzed against 100 mM HEPES-KOH (pH 7.9), 150 mM NaCl, 10% glycerol, 0.5 mM EDTA, 0.1% Nonidet P-40, 2 mm dithiothreitol, in the presence or absence of 50 mM DHT. The Bradford assay was used to determine protein concentration.

**GST pull down assay**

100 picomoles of each protein was made up to 150 μl with pull down buffer (20 mM HEPES pH 7.9; 10% glycerol, 100 mM NaCl; 5 mM MgCl_2_; 0.1% Tween-20; 0.2 mM EDTA pH 8.0; 0.02 mg/ml BSA; 1 mM DTT; 0.2 mM PMSF). 2 picomoles of CHKA (TP307209, Origene) was then added and incubated with gentle shaking at 4 ºC for 2 hr. Following incubation, beads were pelleted and excess buffer was removed. Beads were washed five times with 200 μl pull down buffer and then were resuspended in 20 µl of 2X SDS sample buffer, denatured at 75 ºC for 5 min, pelleted and 20 μl of the supernatant of each sample was analysed by SDS-PAGE.

**Proteolytic Digestion Assay**

Twenty-five picomoles of AR^DBD-LBD^ ± DHT was incubated with 2 picomoles of CHKA (Origene technologies, Rockville, MD) or GST for 90 min. an additional 50 μM DHT was added to the AR^DBD-LBD^ + DHT fraction. Proteolysis was then carried out with 10 ng/μl or 100 ng/μl trypsin in 10 μl final volume of proteolysis buffer (25 mM HEPES (pH 7.9), 10% glycerol, 0.2 mM EDTA, 5 mM MgCl_2,_ 20 mM CaCl_2,_ 60 mM KCl) for 15 min at room temperature. The reaction was stopped with 5X SDS loading buffer and heated for 5 min at 75 ºC. Each sample was analysed by SDS-PAGE followed by western blotting.

**Co-immunoprecipitation**

Co-immunoprecipitation analysis was performed as we described previously ([1](#_ENREF_1)). Two micrograms of AR antibody (Rabbit polyclonal; Santa Cruz Biotechnology N-20, Santa Cruz, CA) per 200 μg cell lysate were used. Protein lysates from cultured cells were prepared and 30 μg protein was subjected to SDS-PAGE and Western blotting was performed. AR and CHKA were detected using AR antibody from Millipore (Rabbit polyclonal PG-21; dilution 1:500) and CHKA antibody from Sigma (Rabbit polyclonal; HPA024153; dilution 1:250).

**Detection of colocalization between the AR and CHKA**

HeLa cells seeded on glass coverslips in DMEM medium containing 5% charcoal-stripped serum (CSS) were transiently co-transfected with the expression vectors coding for *green fluorescent protein* tagged AR (GFP-AR T877A; green) and mCherry-CHKA (red) fusion proteins using a modified calcium phosphate method ([5](#_ENREF_5), [6](#_ENREF_6)). After 48 h, cells were treated either with vehicle or R1881 (1 nM) in 5% CSS containing medium for 2 h. Thereafter, cells were fixed using 4% formaldehyde solution and mounted onto glass slides using ProLong® Gold anti-fade reagent with DAPI (Life Technologies). The intracellular distribution of AR and CHKA was visualised using a fluorescence microscope (Axio Observer.Z1; Carl Zeiss, Jena, Germany) supplemented with the ApoTome device for generation of optical sections. Colocalisation was quantified using BioImageXD software ([7](#_ENREF_7)). At least 10 cells per treatment were quantitatively analysed with 15 z-stack images obtained per cell.

**Western blot analysis**

Samples subjected to SDS-PAGE were transferred to a PVDF membrane (Amersham, Slough, UK) and transfer efficiency was checked with Ponceau red. The membrane was blocked with 5% fat-free milk for 1 hr at room temperature then washed with TBS-T (TBS, 0.05% Tween-20) and incubated with 1:250 diluted anti-CHKA antibody (Rabbit polyclonal; Sigma) or 1: 1000 diluted anti-AR (C19, Rabbit polyclonal; Santa Cruz Biotech.) or anti-AR (Mouse monoclonal; Dako, Carpintaria, CA) antibody overnight at 4 ºC. After washing, the membrane was incubated with goat-anti rabbit HRP antibody for 1 hr at 4 ºC and the presence of CHKA or AR^DBD-LBD^ was determined by chemi-luminescence.

**RNA-sequencing (RNAseq) and comparison with the AR transcriptome**

Total RNA was isolated using the Allprep kit (Qiagen, Hilden, Germany), quantified using Qubit (Life technologies) and quality (RIN>8) was confirmed using the Bioanalyzer 2100 (Agilent). The kit used for the RNA-seq project was the TruSeq® mRNA HT Sample Prep Kit with a 500 ng of total RNA as input. Single-end 40 bp reads generated on the Illumina HiSeq were aligned to the human genome version GRCh37.64 using TopHat v2.0.4 ([8](#_ENREF_8)). Read counts were obtained using HTSeq-count v0.5.3p9 ([9](#_ENREF_9)), normalised and tested for differential gene expression using the Bioconductor package *DESeq* v1.10.1 ([10](#_ENREF_10)). Multiple testing corrections were applied using the Benjamini-Hochberg method ([11](#_ENREF_11)). Genes were selected as differentially expressed such that FDR < 0.05.

To address the global impact of CHKA on the AR transcriptome, the mRNA sequencing data from C4-2 cells was compared with our published androgen-regulated transcriptome dataset (GSE18684) ([12](#_ENREF_12)).

#### Real-time qPCR

C4-2 cells were transiently transfected with the selected siRNA (25 nM) for 48 hr. Cells were then grown in androgen-deprived charcoal treated RPMI medium and treated with R1881 (1 nM) for 24 hr. cDNA synthesis was performed using a High Capacity cDNA Reverse Transcription Kit (Applied Biosystems, Carlsbad, CA). We used taqman quantitative real-time PCR probes (Applied Biosystems) to quantify gene expression changes in TMPRSS2, FKBP5 and NKX3.1 relative to 18S expression, which served as the internal control. AR knockdown was used as the positive control; experiments were carried out with 9 replicates.

**CHKA immunohistochemistry and evaluation**

IHC for CHKA was performed using an avidin-biotin-peroxidase complex (R.T.U. Vectastain Elite ABC Kit [Universal] (Vector Laboratories, Burlingame, CA) (dilution 1:20; Cat. No HPA024153, Atlas Antibodies). Immunoreaction in TMA sections was evaluated for CHKA staining which was predominantly cytoplasmic. Briefly, sections were scored semi-quantitatively as follows: 0: 0% of immunoreactive cells; 1: < 5% of immunoreactive cells; 2: 5-50% of immunoreactive cells and 3: > 50% of immunoreactive cells. The intensity of staining was also scored semi-quantitatively, viz: 0: negative; 1: weak; 2: moderate and 3: strong. The final score was defined as the sum of both parameters (extension and intensity), and grouped as negative (scores 0-3) and positive (scores 4-6). Discordant results in different cores from the same patient were scored as follows: average of extension plus highest intensity score. Statistical analysis was performed using the SPSS statistical software (version 17.0, SPSS Inc., Chicago, IL, USA). Statistical significance was assessed using Pearson's Chi square *(χ*^2^) test, using the *p* < 0.05 as the threshold for significance.

**Immuno-histochemistry for the androgen receptor (AR) and cleaved caspase-3 (CC3)**

Immuno-histochemical staining of paraffin embedded tissues was performed to detect AR and CC3. Sections were stained on a BondMax Autostainer (Leica, Milton Keynes, UK). Antigen retrieval was performed using standard trisEDTA method at 100 ºC for 20 min followed by a 15 min incubation with primary antibodies for AR (Rabbit polyclonal from Santa Cruz, sc-816 at 1:750 dilution) and CC3 (cleaved caspase-3; Rabbit monoclonal; 9664, Cell Signalling Technology) at room temperature, and 8 min incubation with a secondary antibody (biotinylated donkey anti-rabbit; cat. No 711-065-152 from Jackson Immunoresearch, West Grove, PA) using a polymer secondary system (Leica microsystems, Wetzlar, Germany) followed by developing with Diaminobenzidine using enhancer (SP-2001; Vector labs). Haematoxylin counterstaining was performed automatically on the Bond system, and finally, the slides were dehydrated, cleared and mounted using a Leica ST5020 attached coverslipper CV5030 (Leica microsystems). Slides were scanned onto Aperio/SpectrumTM v10.2.2.2317 and analysed using ImageScope (Aperio software v12.0.0.5039). AR and CC3 staining was quantified using an algorithm that identifies nuclei, and computes the number of positively stained nuclei. To identify the epithelial structures for quantification on slides with weak AR staining, serially cut H&E and cytokeratin stained sections were used to pinpoint the exact location for analysis. For each condition at least 400 cells were counted and staining positivity was expressed as the percentage of the total number of cells on the slides (n=>3).

**High content imaging cytometry and apoptosis detection**

For laser scanning cytometry, cells were grown in Ibidi ibiTreat 8 well µ-slides (Ibidi GmbH, Germany), fixed in 4% paraformaldehyde, immuno-stained with an antibody against  AR (DAKO, dilution 1:2500) and detected with  Alexa Fluor® 647 antibody (Life technologies). DNA was counterstained with DAPI.  Fluorescence was determined by quantitative imaging cytometry using an iCys Research Imaging Cytometer (CompuCyte, Cambridge, MA) with iNovator software (CompuCyte).  A scanning protocol for quantification was configured using two channels; 405nm diode laser excitation with blue channel detection (445nm-485nm) for DAPI and HeNe 633 nm laser excitation with long red channel detection (650nm LP) for AR fluorescence.

High resolution scans were acquired using the 60x objective and 0.5mm x-step size. Watershed filters were applied to separate closely spaced events. Nuclear AR fluorescence intensities were determined and expressed as intensities per cell (fluorescence integral).

Apoptosis was quantified using DNA content versus intensity. Apoptotic cells are identified based on their bright fragmented DNA and are easily gated and counted on a scattergram plot of DNA content versus DAPI MaxPixel (brightest pixels).

**Boyden chamber Invasion Assay**

The fluorescence based invasion assay kit was purchased from Cell Biolabs (San Diego, CA) and the assay was performed following the manufacturer’s protocol. Briefly, 48 hr post siRNA transfection, 60,000 C4-2 cells were placed in the upper chamber for 24 hr. RPMI medium with 10% FBS was placed in the lower chamber as a chemo-attractant. Invading cells were stained and optical density was measured at 560 nm as per manufacturer’s instructions.

**Generation of inducible C4-2b cells**

Mammalian inducible expression vector pTRIPZ containing hairpins targeting CHKA and luciferase were obtained from Dharmacon. Three different inducible C4-2b cell clones were generated (sh#1 V2THS-113012, sh#2 V2THS-113013 and sh#3 V2THS-335370) as per manufacturer’s instructions. To optimise the proportion of strongly inducible cells, they were treated with doxycycline (1 µg/ml) for 48 hr to switch on turbo *red fluorescent protein* (tRFP) expression and the top 20% of cells with the strongest expression were sorted by flow cytometry and expanded by culturing in RPMI with 10% FBS and Puromycin (2 µg/ml). These cells were then sorted again to remove any cells with leaky expression of tRFP in the absence of doxycycline. The cells were then expanded and used to initiate tumour xenografts in mice.

#### *Ex vivo* prostate explant culture

Human PCa tissue was collected with informed consent according to the institutional policy. The tissue was cut into 1-2 mm^3^ pieces and grown as explants on collagen cushions kept on steel grids for one week. The tissue was treated as indicated with drugs in RPMI with 10% FBS, 1% penicillin, streptomycin and gentamycin. Collagen cushions, were prepared using 250 μl of collagen mix (rat tail collagen, RPMI medium, FBS and 10X RPMI in the ratio of 7:1:1:1) which was solidified on a nylon membrane. At the end of experiment, the tissue was fixed in formalin for 20 hr and then transferred to 70% ethanol prior to paraffin embedding for immunohistochemistry.

**Matrigel inverted cell invasion**

20,000 GFP positive cells were plated on Collagen I coated membranes (Control Cell Culture Inserts 1.8 µm PET membrane, 40578; BD BioCoat^TM^) in standard culture medium with 10% FBS and placed in a 24 well plate with 1 ml of medium. After 8 hr to allow complete attachment, the medium was removed and membranes were placed on a 20 cm Corning plate.

0.5 ml of cold medium with FBS and 0.5 ml of Matrigel® (Life technologies) were placed on each membrane and allowed to solidify for 1 hr in an incubator at 37 ^o^C with 5% CO_2_. At this time (time-point 0) the membranes were scanned using a confocal Leica Microscope TCS SP5 with 10x objective and 10 nm Z stacks were acquired. Inserts were then placed in a 24 well plate (3524; Costar) and 0.5 ml of medium with 30% FBS was added inside the insert, over the membrane, and 1 ml of medium without FBS, under the membrane, to create an inverted serum gradient. After two days (time-point 1), further confocal images were acquired again and analyzed using Volocity software. The time-point 0 images allowed quantification of a two standard deviation distribution of cells along the Z-axis. The Z centroid values of objects scanned on the second day (time-point 1), outside of that original 2 standard deviation interval in the positive axis were considered Matrigel invading objects. The values of two biological replicates were pooled and the geometric mean and 95% confidence interval calculated.

**Scratch wound assay**

VCaP cells were selected for the scratch wound assay on account of their strong adherence to tissue culture plates. Cells were transfected with 25 nM of the indicated siRNA for 24 hr, by which time nearly confluent layers of VCaP cells had formed in a 24 well tissue culture plate. The scratch was introduced using a wound scratch instrument (Essen Bioscience) with a micropipette tip (1-2 µl capacity). The cells were washed with PBS, given fresh medium and treated with the indicated drugs. The culture plate was placed inside the Incucyte instrument and monitored for the required time.

**Principal component analysis (PCA)**

The ^1^H NMR spectral data (LNCaP control cell samples = 12, treated samples = 9) from 0.50 to 4.20 ppm were binned at 0.01 ppm intervals using Bruker AMIX software. Only positive intensity values were considered and individual bin intensity was normalized to the total intensity of the bins in the 0.50 to 4.20 ppm range. The normalised binned data were exported to a Microsoft Excel spread sheet and the SIMCA13.0 (Umetrics) software package was used for Principal Component Analysis (PCA). Each point in the PCA scores plot represents a sample and they were classified according to the formation of clusters in the plot. Samples with similar ^1^H NMR spectral patterns will form as a cluster in the plot. The loadings plot shows the bins (in this case the chemical shifts of the metabolites) which result in the separation or classification of samples in the scores plot. PCA was performed using the mean centred metabolite data. Subsequently orthogonal projection to latent structure – discriminant analysis (OPLS-DA) was conducted using the mean centred data as the X-matrix (each row representing a sample and each column a metabolite) and class information as the Y-matrix, to identify metabolites that discriminate between the two classes.

**SUPPLEMENTARY FIGURE LEGENDS**

**Supplementary Figure 1.** Interrogation of AR-regulated kinases in PCa related to figure 1. **A**) Venn diagram for LNCaP data showing the cross-platform validation on Illumina Human6v2 Bead-arrays and the Openarray kinome panel for 86 kinases represented on both platforms. **B**) Bar chart showing occurrence of AR binding sites within genes encoding androgen regulated kinases, and within 5 kb or 25 kb of those genes in LNCaP cells. **C-D**) Heatmaps generated through supervised clustering showing the expression of androgen-regulated kinases in ([13](#_ENREF_13)) (Fig. S1C) and ([14](#_ENREF_14)) (Fig. S1D). Arrow indicates CHKA in the gene expression heat map. **E**) Heatmap of transcript expression of kinases in Degarelix treated (n=15) and control tumours (n=19) in patients. **F**) Cytoscape analysis of androgen-regulated kinases, showing the direct interactions of the core 49 androgen-regulated kinases (union of HPRD, BIOGRID and KEGG direct interactions generated using BioNetBuilder). Kinases up-regulated by androgens are coloured red and kinases down-regulated by androgens are coloured green. Nodes are genes and lines connecting the nodes denote interactions. PCR=Polymerase chain reaction; AR=Androgen receptor;

**Supplementary Figure 2.** CHKA is an AR target in human PCa; related to figure 1. **A**) ChIP-seq analysis from LNCaP, 22Rv1 and VCaP cell lines showing the genomic locus of the CHKA gene and the AR binding sites, together with enriched H3K4me1 and H3K4me3 marks. **B**) ChIP-seq analysis of CRPC tissues from two different patients showing the genomic locus of the CHKA gene and the binding sites of AR along with H3K4me1 and H3K4me3 marks. **C**) Time course of CHKA transcript induction in response to R1881 in LNCaP and VCaP cells; log2 expression values are shown. **D**) Western blot showing the protein levels of AR and CHKA in LNCaP shScr/shAR expressing cells ([15](#_ENREF_15)) grown in hormone-depleted medium and treated with R1881 (1 nM) and/or doxycycline (1 μg/ml) for 72 hr; β-actin is a loading control. **E**) Western blot showing CHKA expression in LNCaP and C4-2 cells transfected with siRNA as indicated for 96 hr; β-actin is the loading control. **F**) Intensity score of CHKA protein expression in non-neoplastic (NN), prostate intraepithelial neoplasia (PIN) and prostate tumour tissue (TT); significance calculated by Chi-square test. **G)** Co-immuno precipitation showing interaction of endogenous AR and CHKA. LNCaP cells were grown in hormone-depleted medium and cell were harvested 48 hr with treatment or no treatment ± DHT (10 nM). Nuclear fraction was incubated with indicated antibodies targeting either AR or CHKA followed by western blotting using the AR antibody. **H**) Protease protection assay of AR^DBD-LBD^. His-tagged AR^DBD-LBD^ was expressed and purified without DHT and subjected to proteolytic digestion with 10 ng/μl trypsin ± pre-incubation with CHKA and ± DHT. Full-length (band 1a) receptor polypeptide or proteolytic fragments (bands 1b, 2, 3 and 4) were then detected using an anti-AR C19 antibody. ChIP=Chromatin immunoprecipitation; AR=Androgen receptor; CHKA=Choline kinase alpha; Scr=Scrambled, sh=short hairpin; si=small interferaing; NN=Non-neoplastic; PIN=Prostate intraepithelial neoplasia; TT=Tumor tissue; IP=Immunoprecipitation; DMSO=Dimethyl sulfoxide; Cont=control; DHT=Dihydrotestosterone.

**Supplementary Figure 3.** CHKA inhibition decreases AR activity, related to figure 3. **A**) Luciferase assay of the MMTV-Luc reporter in C4-2 cells. Transfected cells were treated for 48 hr with the drugs indicated; bars show mean ± SD (n=3). P values by two-sided Student’s t-test. **B**) Western blot of AR and CHKA proteins in C4-2 cells treated with R1881 (1 nM) and CHKAi (1 μM) for 24 hrs, β-actin is a loading control. **C**) Upper panel: Photomicrographs showing nuclear AR (red) content of LNCaP cells treated with R1881 (1 nM), bicalutamide and CHKAi (both at 1 μM) for 24 hr (upper panel). Lower panel: Quantification of high-content imaging cytometry from LNCaP cells treated with R1881 (1 nM), bicalutamide (1 μM) and CHKAi (1 μM) (lower panel); bars show mean ± SD (n=3). Scale bar=50 µm. **D**) qPCR of TMPRSS2, FKBP5 and NKX3.1 gene transcripts from C4-2 transfected with siScr, siCHKA or siAR for 72 hr and treated ±R1881 for additional 24 hr; bars show mean ± SD (n=9). MMTV=Mouse mammary tumor virus; Bic=Bicalutamide; Enz=Enzalutamide; CHKAi=Choline kinase alpha inhibitor.

**Supplementary Figure 4.** Inhibition of CHKA decreases PCa cell growth; related to figure 4. **A**) Growth of C4-2 cells treated with bicalutamide or CHKAi (10 µM) or transfected with siRNA targeting AR or CHKA; line show mean ± SD (n=6). **B-C**) MTS cell viability assay of PCa cell lines namely DUCaP, LNCaP-Bic ([16](#_ENREF_16)) and 22Rυ1, transiently transfected with 25 nM siNT, siCHKA or siAR, showing their respective response to androgen R1881 (1 nM) treatment for 5 days; bars show mean ± SD (n=3). P values by two-sided Student’s t-test. **D**) MTS assay of the LNCaP-LN3 cell line ([17](#_ENREF_17)) treated with R1881 (1 nM) and the indicated concentration of CHKAi; bars show mean ± SD (n=3). P values by two-sided Student’s t-test. **E**) MTS cell viability assay in the indicated cell lines treated with R1881 (1 nM), bicalutamide (1 µM) and CHKAi (1 µM); bars show mean ± SD (n=3). **F-G**) Clonogenic cell survival assay for the **F**) DUCaP, and **G**) LNCaP-LN3 cell lines treated for 14 days with bicalutamide, enzalutamide and CHKAi (all at 10 µM); P values by two-sided Student’s t-test. AD=androgen-dependent, AI=androgen independent; bars show mean ± SD (n=3). **H**) MTS assay of C4-2 cells treated with R1881 (1 nM) and the indicated concentration of PCho and/or PdCho; bars show mean ± SD (n=3). P values by two-sided Student’s t-test. **I**) High content cytometry based apoptotic assay showing fold change in the number of apoptotic C4-2 cells observed under the conditions indicated. Cell were treated for 24 hr; bars show ± SD (n=3). NT=Non-targeting; CHKA=Choline kinase alpha; AR=Androgen receptor; Bic=Bicalutamide; Enz=Enzalutamide; AD=Androgen-dependent; AI=Androgen-independent.

**Supplementary Figure 5.** CHKA inhibition decreases tumour cell growth and invasion; related to figure 5. **A**) Mouse images showing turbo-red fluorescent protein expression in C4-2b tumour xenografts treated with doxycycline for 2 weeks. Fluorescence was measured using Xenogen camera. **B-C**) Intensity scores of CHKA **B**) and AR **C**) in the xenograft tissue derived from mice ± dox (n=3 from each group). The intensity was classified as weak, moderate or strong (P<0.05). **D**) Barplot of trans-well migration assay showing the relative invasive ability of C4-2 cells after AR or CHKA knockdown with 25 nM siRNA; bars show mean ± SD (n=3). P values by two-sided Student’s t-test. Dox=Doxycycline; sh=short hairpin RNA; Luc=Luciferase; CHKA=Choline kinase alpha; AR=Androgen receptor; NT=Non-targeting siRNA.

**Supplementary Figure 6.** Androgen-regulated kinases are modulated *in vivo*; related to figure 1. **A**) Barplots showing transcript expression of androgen up-regulated kinases in tissues from men with untreated PCa and degarelix treated PCa (p<0.05 for all genes shown). **B**) Barplots showing transcript expression of androgen down-regulated kinases in tissues from men with untreated PCa and degarelix treated PCa (p<0.05). Boxplots show data spread, boxes show IQR, error bars show 95% CI and data points show outlier values. IQR=Interquartile ratio.

**Supplementary Table 1** **A-B**) Changes in the transcript level of androgen-regulated kinases as a function of time, identified both on the Illumina beadArray and Openarray kinome panel.

**Supplementary Table 2:** Table of the results of the effects of CHKA on biochemical recurrence free survival using Cox regression, first column shows the additional clinical variable included in each model. * The number of cases is the same to ensure comparability between models and is therefore all the cases not missing survival and clinical information.

| Variable adjusted for | N* (events) | HR (95% CI) |
| --- | --- | --- |
| CHKA only | 228 (45) | 1.99 (1.09, 3.61) |
| Gleason | 228 (45) | 1.76 (0.95, 3.28) |
| Tumour Stage | 228 (45) | 1.88 (1.03, 3.42) |
| Age | 228 (45) | 2.00 (1.10, 3.63) |
| PSA | 228 (45) | 2.01 (1.10, 3.64) |

* The number of cases is the same to ensure comparability between models and is therefore all the cases not missing survival and clinical information.

Table of the results of the effects of CHKA on biochemical recurrence free survival using Cox regression, first column shows the additional clinical variable included in each model. HR=Hazard ratio; CI=Confidence interval; CHKA=Choline kinase alpha; PSA=Prostate specific antigen.

**Supplementary Table 3:** Quantitative colocalisation analysis of CHKA and AR

| Ligand | **Manders’ coefficients^a^** | | **R(obs)^b^** | **R(rand)^c^ (mean±SD)** | **p-value^d^** |
| --- | --- | --- | --- | --- | --- |
|  | M1 | M2 |  |  |  |
| Vehicle | 0.866 | 0.842 | 0.728 | 0.025**±**0.003 | 1.00 |
| R1881 | 0.861 | 0.620 | 0.565 | 0.037**±**0.002 | 1.00 |

(a) Manders’ coefficients for channel 1 (green) and channel 2 (red) varying between 0 and +1, with 0 for no overlap and +1 for perfect overlap.

(b) Pearson’s correlation coefficient for the two selected channels varying between -1 and +1, with -1 for total negative correlation, 0 for random correlation, and +1 for perfect correlation.

(c) Pearson’s correlation coefficient for channel 1 against a number of randomized channel 2 images.

(d) Costes’ randomization p-value of ≥0.95 indicates significant true colocalisation.

**Supplementary table 4:** Table for all the genomic data used in the manuscript including GEO accessions or web links. The probeset used in each data set also included.

| GEO_Accession / weblink | PMID | Figures using data |
| --- | --- | --- |
| GSE18684 | 21602788 | 1A |
| GSE28126 | 21602788 | 1B |
| GSE28219 | 23260764 | 1B |
| GSE14092 | 20478527 | 1B |
| http://research.dfci.harvard.edu/brownlab/datasets/ | 19632176 | 1B |
| GSE3325 | 16286247 | 1C |
| GSE35988 | 22722839 | 1D |
| Data will be uploaded to GEO following publication | Shaw G, et al (manuscript in preparation) | 1H |
| data requested from authors | 15067324 | 1H |
| GSE63700 | This manuscript | 1F-L |
| GSE3325 | 16286247 | Suppl.Fig.1C |
| GSE35988 | 22722839 | Suppl.Fig.1D |
| Data will be uploaded to GEO following publication | Shaw G, et al (manuscript in preparation) | Suppl.Fig.1E |
| GSE28126 | 21602788 | Suppl.Fig.2A |
| GSE28219 | 23260764 | Suppl.Fig.2B |
| GSE18684 | 21602788 | Suppl.Fig.2C |

**SUPPLEMENTARY REFERENCES**

1. Asim M, Hafeez BB, Siddiqui IA, Gerlach C, Patz M, Mukhtar H, et al. Ligand-dependent corepressor acts as a novel androgen receptor corepressor, inhibits prostate cancer growth, and is functionally inactivated by the Src protein kinase. J Biol Chem. 2011;286(43):37108-17.

2. Zhang J, Thomas TZ, Kasper S, Matusik RJ. A small composite probasin promoter confers high levels of prostate-specific gene expression through regulation by androgens and glucocorticoids in vitro and in vivo. Endocrinology. 2000;141(12):4698-710.

3. Khan N, Asim M, Afaq F, Abu Zaid M, Mukhtar H. A novel dietary flavonoid fisetin inhibits androgen receptor signaling and tumor growth in athymic nude mice. Cancer Res. 2008;68(20):8555-63.

4. Berrevoets CA, Doesburg P, Steketee K, Trapman J, Brinkmann AO. Functional interactions of the AF-2 activation domain core region of the human androgen receptor with the amino-terminal domain and with the transcriptional coactivator TIF2 (transcriptional intermediary factor2). Mol Endocrinol. 1998;12(8):1172-83.

5. Moehren U, Papaioannou M, Reeb CA, Grasselli A, Nanni S, Asim M, et al. Wild-type but not mutant androgen receptor inhibits expression of the hTERT telomerase subunit: a novel role of AR mutation for prostate cancer development. Faseb J. 2008;22(4):1258-67.

6. Hessenkemper W, Roediger J, Bartsch S, Houtsmuller AB, van Royen ME, Petersen I, et al. A natural androgen receptor antagonist induces cellular senescence in prostate cancer cells. Mol Endocrinol. 2014;28(11):1831-40.

7. Kankaanpaa P, Paavolainen L, Tiitta S, Karjalainen M, Paivarinne J, Nieminen J, et al. BioImageXD: an open, general-purpose and high-throughput image-processing platform. Nat Methods. 2012;9(7):683-9.

8. Trapnell C, Pachter L, Salzberg SL. TopHat: discovering splice junctions with RNA-Seq. Bioinformatics. 2009;25(9):1105-11.

9. Anders S, Pyl PT, Huber W. HTSeq-a Python framework to work with high-throughput sequencing data. Bioinformatics. 2014.

10. Anders S, Huber W. Differential expression analysis for sequence count data. Genome Biol. 2010;11(10):R106.

11. Benjamini Y, Hochberg Y. Controlling the False Discovery Rate - a Practical and Powerful Approach to Multiple Testing. J Roy Stat Soc B Met. 1995;57(1):289-300.

12. Massie CE, Lynch A, Ramos-Montoya A, Boren J, Stark R, Fazli L, et al. The androgen receptor fuels prostate cancer by regulating central metabolism and biosynthesis. Embo J. 2011;30(13):2719-33.

13. Varambally S, Yu J, Laxman B, Rhodes DR, Mehra R, Tomlins SA, et al. Integrative genomic and proteomic analysis of prostate cancer reveals signatures of metastatic progression. Cancer Cell. 2005;8(5):393-406.

14. Grasso CS, Wu YM, Robinson DR, Cao X, Dhanasekaran SM, Khan AP, et al. The mutational landscape of lethal castration-resistant prostate cancer. Nature. 2012;487(7406):239-43.

15. Cheng H, Snoek R, Ghaidi F, Cox ME, Rennie PS. Short hairpin RNA knockdown of the androgen receptor attenuates ligand-independent activation and delays tumor progression. Cancer Res. 2006;66(21):10613-20.

16. Hobisch A, Fritzer A, Comuzzi B, Fiechtl M, Malinowska K, Steiner H, et al. The androgen receptor pathway is by-passed in prostate cancer cells generated after prolonged treatment with bicalutamide. Prostate. 2006;66(4):413-20.

17. Pettaway CA, Pathak S, Greene G, Ramirez E, Wilson MR, Killion JJ, et al. Selection of highly metastatic variants of different human prostatic carcinomas using orthotopic implantation in nude mice. Clin Cancer Res. 1996;2(9):1627-36.
